# Symptom Burden and Healthcare Resource Use in Patients With Claudin 18.2–Positive, HER2-Negative, Locally Advanced Unresectable or Metastatic Gastric or Gastroesophageal Junction Adenocarcinoma: A Retrospective Review

**DOI:** 10.1177/11795549261430041

**Published:** 2026-03-29

**Authors:** Matheus Sewastjanow-Silva, Mok Oh, Lawrence Chang, Arijit Ganguli, Jane E Rogers, Rebecca E Waters, Ernesto Rosa Vicentini, Kohei Yamashita, Todor I Totev, Eric Q Wu, Hongbo Yang, Kenneth Iwata, Jaffer A Ajani

**Affiliations:** 1 Department of Gastrointestinal Medical Oncology, The University of Texas MD Anderson Cancer Center, Houston, TX, USA; 2 Astellas Pharma Global Development, Inc., Northbrook, IL, USA; 3 Pharmacy Clinical Programs, The University of Texas MD Anderson Cancer Center, Houston, TX, USA; 4 Department of Anatomical Pathology, The University of Texas MD Anderson Cancer Center, Houston, TX, USA; 5 Analysis Group, Boston, MA, USA

**Keywords:** Adenocarcinoma, claudin, gastric cancer, gastroesophageal junction, healthcare utilization

## Abstract

**Background:**

Control of disease-related symptoms is a goal of chemotherapy for patients with locally advanced (LA) unresectable or metastatic gastric/gastroesophageal junction (mG/GEJ) adenocarcinoma. This study describes disease-related symptoms and healthcare resource utilization (HRU) in this population.

**Methods:**

A retrospective review of adult patients with claudin 18.2–positive (CLDN18.2+), human epidermal growth factor receptor 2–negative (HER2−), LA unresectable or mG/GEJ adenocarcinoma was performed. Outcomes were assessed from the index date (date of diagnosis) through the follow-up end date (earliest of first-line treatment discontinuation, last follow-up visit, death, or 1 year after index date).

**Results:**

Sixty-two patients were included in the analysis (mean age, 61.3 years; 54.8% male; 88.7% White; 67.7% had gastric primary tumors; 75.8% had peritoneal metastases; 98.4% received first-line treatment [mean time from diagnosis to treatment initiation, 37.0 days]). All patients reported ⩾1 disease-related symptom (mean = 7.2) at the index date. The most common symptoms at the index date were weight loss (74.2%), abdominal pain/stomach pain (66.1%), anemia/weakness (61.3%), poor appetite (56.5%), and epigastric pain (50.0%). Of the 21 patients evaluated at the 6-month follow-up, 95.2% reported ⩾1 disease-related symptom. The greatest changes were seen for weight loss (0.0% at 6 months vs 74.2% at the index date), epigastric pain (9.5% vs 50.0%), poor appetite (23.8% vs 56.5%), reflux (4.8% vs 35.5%), early satiety (0.0% vs 29.0%), and abdominal pain/stomach pain (38.1% vs 66.1%). A mean of 3.4 outpatient visits per patient per month was reported (mean follow-up, 6.5 months), 21.0% of patients had an inpatient admission, and 35.5% had an emergency department visit.

**Conclusions:**

This study demonstrates substantial disease-related symptom burden and high HRU for patients with CLDN18.2+, HER2−, LA unresectable or mG/GEJ adenocarcinoma.

## Introduction

Gastric and gastroesophageal junction (G/GEJ) cancers comprise approximately 6% to 9% of all new cancer cases worldwide and account for approximately 8% to 13% of all deaths due to cancer.^1^ Locally advanced (LA) unresectable or metastatic G/GEJ (mG/GEJ) adenocarcinoma is often incurable.^2^ Disease-related symptoms of LA unresectable or mG/GEJ adenocarcinoma can be intense, increase in severity toward the end of life, and adversely affect patients’ daily living and quality of life.^3-5^ Negative effects on energy level (*i.e.*, fatigue, tiredness, or drowsiness) and poor appetite are among the most prevalent and severe G/GEJ cancer disease-related symptoms reported.^3,5,6^ Fatigue related to mG/GEJ cancer has been reported to increase in severity during the last 6 months of life.^3^ Poor appetite in patients with G/GEJ cancer is associated with weight loss.^3^ Greater weight loss in patients with gastric cancer is associated with worse survival outcomes.^7,8^

Chemotherapy is standard of care for patients with LA unresectable or mG/GEJ adenocarcinoma and is used to delay disease progression and control disease-related symptoms.^3^,^9-11^ The goal of disease-related symptom control with chemotherapy is to help patients better tolerate continued cancer treatment and improve their quality of life.^3^,^9-11^ Although chemotherapy has been shown to improve survival rates for metastatic gastric cancer, disease-related symptom control remains an unmet need.^3,8,9^ Within 12 weeks of starting first-line chemotherapy, more than 50% of patients have been reported to experience weight loss.^8^ At 1 year following initiation of chemotherapy for treatment of gastrointestinal malignancies, approximately 5% of patients continue to report pain.^12^ Chemotherapy has also been shown to have no effect on reducing the severity progression of drowsiness related to gastric cancer.^3^ In addition to the modest benefit seen for disease-related symptom control with chemotherapy for G/GEJ cancer, the economic impact of disease-related symptom treatment can be substantial, although limited reports are available.^9,13,14^ A retrospective analysis of claims data from 2007 to 2012 reported that patients who had gastric cancer incurred more than 10 times the healthcare costs compared with patients without cancer; patients with advanced disease incurred the highest costs.^15^ A later retrospective claims analysis, performed from October 2011 through July 2017, reported a high economic burden associated with advanced or metastatic GEJ cancer.^13^ In that study, 35% of patients who received first-line treatment subsequently received second-line treatment, while 65% did not, highlighting an unmet need for novel treatments and the frailty of these patients, many of whom are unable to tolerate chemotherapy. The overall healthcare costs for a patient with gastric cancer in Taiwan or the United States were found to be greater than the costs for a patient with colorectal, liver, or lung cancer.^9^ The combined mean costs of outpatient visits for patients with human epidermal growth factor receptor 2 (HER2)–negative (HER2−) G/GEJ cancer are reported to range from approximately $11 000 to $13 000 per patient per month (PPPM).^14^

A thorough understanding of patients’ disease-related symptom burden and healthcare resource utilization (HRU) is required to better characterize disease burden and support current and future research and treatment strategies. The objective of this retrospective study was to describe the real-world disease-related symptoms of patients with claudin 18.2–positive (CLDN18.2+), HER2−, LA unresectable or mG/GEJ adenocarcinoma, and the HRU in this population.

## Methods

### Study design

**Figure 1.**
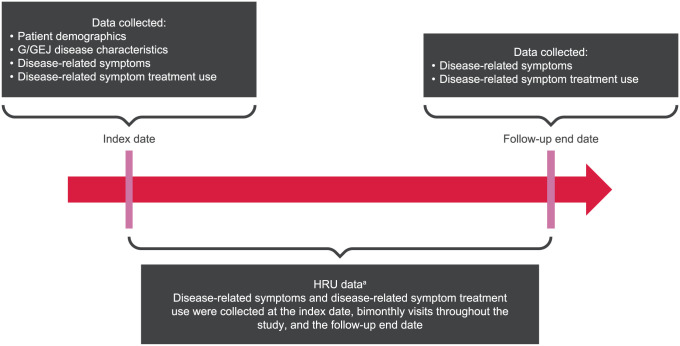
Data collection time points of interest and data collected. Abbreviations: G/GEJ, gastric/gastroesophageal junction; HRU, healthcare resource utilization. ^a^Includes the number of visits, length of stay, and reason for visit.

A retrospective, noninterventional chart review of the medical records of adult patients with CLDN18.2+, HER2−, LA unresectable or mG/GEJ adenocarcinoma evaluated at MD Anderson Cancer Center (MDACC) in Houston, Texas, from December 14, 2021, to May 13, 2022, was performed. Disease-related symptoms, HRU, and associated disease-related symptom treatment data were collected at the index date, at the follow-up end date, and at visits that occurred every 2 months during year 1 of first-line treatment (Figure 1). The index date was defined as the date of diagnosis of LA unresectable or mG/GEJ adenocarcinoma. The follow-up end date was defined as the earliest date of the following: first-line treatment discontinuation, last follow-up visit, death, or 1 year after the index date. The study protocol was approved by the Institutional Review Board of the University of Texas MDACC (protocol: 2020-1316; approval date: January 7, 2021), and all participants provided written informed consent to the study’s use of their clinical data. The study followed the ethical standards of the Declaration of Helsinki of 1975, as revised in 2024. The reporting of this study conforms to the STrengthening the Reporting of OBservational studies in Epidemiology (STROBE) guidelines.^16^

### Patients

Patients included in the analysis were aged ⩾ 18 years when diagnosed with LA unresectable or mG/GEJ adenocarcinoma, had tumor tissue samples identified as CLDN18.2+ (defined as ⩾ 75% of tumor cells showing moderate [2+] or strong [3+] membrane immunohistochemistry staining with antibody clone 43-14A, consistent with other publications^17-20^) and HER2−, had a complete or near-complete medical record for review, and had ⩾3 months of follow-up, unless the patient died or discontinued first-line treatment within the first 3 months of the study period. Patients who had tumor tissue samples identified as HER2+ were not included in the analysis. All patient details have been de-identified.

### Data collected and study endpoints

Patient and disease characteristics were collected at the index date, and data on a wide variety of disease-related symptoms, symptom severity, and treatment use for disease-related symptoms were recorded at each visit. Because this was a retrospective chart review, symptom reporting and categorization were at the treating physician’s discretion.

The study had 3 coprimary endpoints. The first coprimary endpoint was to summarize disease-related symptoms at the index date, including the proportion of patients who had at least 1 disease-related symptom, the proportion of patients who had at least 1 moderate or severe disease-related symptom, the proportion of patients who had at least 1 severe disease-related symptom, the proportion of patients with each disease-related symptom, and the total number of disease-related symptoms. The second coprimary endpoint was to summarize the disease-related symptoms that occurred between the index date and the follow-up end date, including the proportion of patients who had at least 1 disease-related symptom, the proportion of patients who had at least 1 moderate or severe disease-related symptom, the proportion of patients who had at least 1 severe disease-related symptom, the proportion of patients with each disease-related symptom, the total number of disease-related symptoms, and the proportion of patients who received disease-related symptom treatment. The final coprimary endpoint was to evaluate the change in disease-related symptoms from the index date to the follow-up end date, including the proportion of patients with at least 1 new disease-related symptom, the proportion of patients with a change in associated disease-related symptom treatment use, and the mean change in total number of symptoms between visits. Symptom data were collected at the index date prior to the initiation of chemotherapy to better differentiate disease-related symptoms from treatment-related symptoms. Symptom data collected at each visit following the index date may include both disease-related symptom data and treatment-related symptom data.

Secondary endpoints summarized patient and disease characteristics and HRU data. Patient characteristics included demographics, comorbidities, and mortality status at the time of data collection. Patient disease characteristic data included the American Joint Committee on Cancer (AJCC)^21^ disease stage at the time of LA unresectable or mG/GEJ adenocarcinoma diagnosis, Eastern Cooperative Oncology Group (ECOG) performance status,^22^ sites of metastasis, tumor type at the time of LA unresectable or mG/GEJ adenocarcinoma diagnosis, the mean time (in days) from initial diagnosis of LA unresectable or mG/GEJ adenocarcinoma to initiation of first-line treatment, CLDN18.2 expression, and programmed cell death ligand 1 (PD-L1) status. Summarized HRU data included the mean number of HRU visits (outpatient and emergency department visits and inpatient admissions) PPPM, the reasons for HRU visits and admissions, if available, and length of hospital stay.

### Statistical analysis

Continuous variables were described with mean and standard deviation (SD). Categorical variables were described with frequency counts and percentages. A response was required for all fields. Answers to questions that were deemed not applicable were coded as missing (*e.g.*, information on initial G/GEJ adenocarcinoma diagnosis was skipped if a prior response indicated that the first diagnosis was LA unresectable or mG/GEJ adenocarcinoma). An option to select “unknown” was provided for cases in which information was unavailable. The frequency of “unknown” responses was reported for these variables. The total number of patients for whom a question was applicable was used to determine percentages. The percentage of “unknown” responses was calculated for questions where “unknown” was provided as a response option. The length of a month was defined as 30.4375 days, calculated as 365.25 (the number of days in a year accounting for a leap year every 4 years) divided by 12 months. The 15th of the month was used for date variables when the month, but not the exact date of an event, was known.

## Results

### Patient disposition

Of the 304 patients with tumor tissue samples in the tissue biorepository, 44% had samples that were CLDN18.2+.^23^ Sixty-three patients who had CLDN18.2+ tumors met the study criteria for inclusion; 62 patients were included in the analysis. One patient was excluded for starting treatment at another institution 49 days before transferring to MDACC and receiving a diagnosis of LA unresectable or mG/GEJ adenocarcinoma.

### Patient demographics and clinical characteristics

The mean (SD) patient age was 61.3 (10.8) years; 54.8% of patients were male and 88.7% were White (Table 1). Overall, 83.9% of patients had commercial/private insurance coverage, and 17.7% had Medicare. All patients had at least 1 comorbidity; the most common comorbidities were hypertension (45.2%), gastroesophageal reflux disease (30.6%), and anemia (27.4%). Most patients (72.6%) were deceased at the time of data collection.

**Table 1.** Patient and disease characteristics at the index date and initiation of first-line treatment.

Characteristic	All patients N = 62
Age (years), mean (SD)	61.3 (10.8)
Male, n (%)	34 (54.8)
Race/ethnicity, n (%)^a^	
White	55 (88.7)
Black or African American	1 (1.6)
Asian	1 (1.6)
Unknown/not sure	1 (1.6)
Hispanic or Latino	12 (19.4)
Weight (kg), mean (SD)	79.6 (18.4)
Insurance type, n (%)^a^	
Commercial/private insurance	52 (83.9)
Medicare	11 (17.7)
No health insurance	1 (1.6)
Patients with at least 1 comorbidity, n (%)	62 (100.0)
Comorbidities, n (%)	
Hypertension	28 (45.2)
GERD	19 (30.6)
Anemia	17 (27.4)
Hyperlipidemia	10 (16.1)
Pulmonary disease other than COPD	10 (16.1)
Diabetes	9 (14.5)
Other cancer^b^	8 (12.9)
Osteoarthritis	6 (9.7)
Anxiety	5 (8.1)
Cardiovascular disease	4 (6.5)
COPD	3 (4.8)
Atrial fibrillation	2 (3.2)
Other infectious disease	2 (3.2)
CHF	1 (1.6)
CAD	1 (1.6)
Electrolyte disorder	1 (1.6)
Hepatitis	1 (1.6)
Other arrhythmia	1 (1.6)
Other^c^	15 (24.2)
Mortality status at time of data collection, n (%)	
Deceased	45 (72.6)
Alive	14 (22.6)
Unknown/not sure	3 (4.8)
Patients initially diagnosed at LA unresectable or metastatic stage, n (%)	
Yes	58 (93.5)
No^d^	4 (6.5)
Tumor location, n (%)	
Stomach	42 (67.7)
GEJ	20 (32.3)
Tumor type, n (%)	
Signet ring cell carcinoma	32 (51.6)
Intestinal	11 (17.7)
Diffuse	6 (9.7)
Mixed	1 (1.6)
Other^e^	10 (16.1)
Unknown/not sure	2 (3.2)
AJCC TNM disease stage IV, n (%)	62 (100.0)
Metastasis site, n (%)	
Peritoneum	47 (75.8)
Distant lymph nodes	13 (21.0)
Liver	9 (14.5)
Lung	6 (9.7)
Bone	4 (6.5)
Other gastrointestinal	4 (6.5)
Diaphragm	2 (3.2)
Abdominal wall	1 (1.6)
Nervous system	1 (1.6)
Thorax	1 (1.6)
Other^f^	4 (6.5)
CLDN18.2 expression level %, mean (SD)	93.8 (8.1)
ECOG performance status as of treatment initiation for LA unresectable or mG/GEJ adenocarcinoma, n (%)	
0	3 (4.8)
1	57 (91.9)
2	2 (3.2)
Time from initial diagnosis of LA unresectable or mG/GEJ adenocarcinoma to initiation of first-line treatment (days),^g^ mean (SD)	37.0 (51.0)
All patients who received first-line treatment,^h^ n (%)	61 (98.4)
Treatment duration (months), mean (SD)	5.4 (3.7)
Patients who discontinued first-line treatment,^h^ n (%)	47 (77.0)
Treatment duration (months), mean (SD)	4.9 (2.5)

Abbreviations: AJCC, American Joint Committee on Cancer; CAD, coronary artery disease; CHF, congestive heart failure; CLDN18.2, claudin 18.2; COPD, chronic obstructive pulmonary disease; EBV, Epstein-Barr virus; ECOG, Eastern Cooperative Oncology Group; GEJ, gastroesophageal junction; GERD, gastroesophageal reflux disease; G/GEJ, gastric/gastroesophageal junction; LA, locally advanced; mG/GEJ, metastatic gastric/gastroesophageal junction; SD, standard deviation; TNM, tumor-node-metastasis.

a Responses are not mutually exclusive.

b Not collected on the electronic case report form.

c Other concurrent disease conditions included dyslipidemia, depression, psoriasis, rheumatoid arthritis, gastric ulcer, hypothyroidism, Meniere’s disease, and polymyalgia rheumatica.

d Diagnosed at an earlier stage; 1 patient with an initial G/GEJ adenocarcinoma diagnosis different from LA unresectable or metastatic diagnosis was initially diagnosed at AJCC TNM disease stage IIA; 3 patients with an initial G/GEJ adenocarcinoma diagnosis different from LA unresectable or metastatic diagnosis had an unknown/not sure initial AJCC TNM disease stage. The stomach was the initial tumor location for all 4 patients.

e Includes poorly differentiated adenocarcinoma; invasive, poorly differentiated adenocarcinoma; invasive, poorly differentiated EBV-associated carcinoma; moderately to poorly differentiated adenocarcinoma; and invasive, moderately to poorly differentiated adenocarcinoma.

f Includes omentum and ovary.

g Includes patients with known first-line treatment initiation date; calculated as the follow-up end date minus the start date plus 1.

h One patient did not start first-line treatment.

Of the 62 patients included in the analysis, 93.5% were initially diagnosed at the LA unresectable or metastatic stage; 67.7% had gastric primary tumors. Signet ring cells were observed in most patients’ tumors (51.6% signet ring cell carcinoma). All patients had AJCC tumor-node-metastasis (TNM) disease stage IV at the time of LA unresectable or mG/GEJ adenocarcinoma diagnosis. The mean (SD) number of metastatic sites was 1.5 (0.8), and the peritoneum (75.8%) was the most common site of metastasis. Most patients (91.9%) had an ECOG performance status of 1 as of treatment initiation for LA unresectable or mG/GEJ adenocarcinoma. First-line treatment was received by 98.4% of patients and was initiated at a mean (SD) of 37.0 (51.0) days after initial diagnosis of LA unresectable or mG/GEJ adenocarcinoma.

### Patient-reported disease-related symptoms

**Figure 2.**
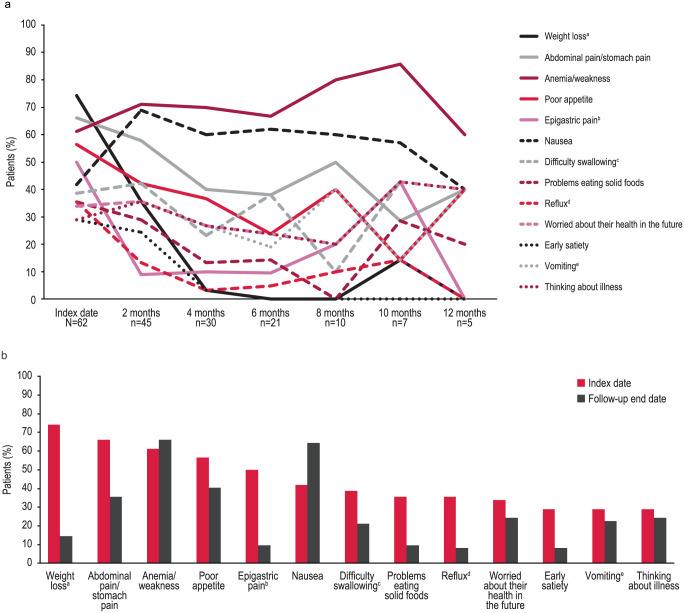
Change in disease-related symptoms reported by ⩾25% of patients at the index date up to 12 months (a) and at the index date and follow-up end date (b). ^a^Loss of ⩾5% since the previous visit. ^b^Epigastric pain was a category separate from abdominal pain. ^c^Dysphagia. ^d^Acid or bile coming into mouth. ^e^With or without blood.

The mean (SD) duration of follow-up for symptom assessment was 6.1 (3.2) months. All patients had at least 1 disease-related symptom at the index date, with a mean (SD) of 7.2 (3.3) disease-related symptoms reported per patient (Table 2). At least 1 moderate or severe symptom was reported by 77.4% of patients at the index date. The most common disease-related signs/symptoms reported at the index date were weight loss (74.2%), abdominal pain/stomach pain (66.1%), anemia/weakness (61.3%), poor appetite (56.5%), and epigastric pain (50.0%; Figure 2). Other disease-related symptoms reported by ⩾25% of patients at the index date were nausea, difficulty swallowing (dysphagia), problems eating solid foods, reflux (acid or bile coming into mouth), worried about their health in the future, early satiety, vomiting (with or without blood), and thinking about illness. Anemia/weakness and nausea were the most frequently observed symptoms between the 2-month visit and the follow-up end date (Figure 2a). The severity level reported for anemia/weakness, nausea, abdominal pain/stomach pain, and poor appetite remained unchanged at the follow-up end date compared with the index date for a majority of patients (81.5%, 68.4%, 76.5%, and 73.7%, respectively; Table 3). In addition, at least 1 new disease-related symptom was reported by 16.1% of patients at the follow-up end date.

**Table 2.** Disease-related symptoms experienced by patients overall.

Disease-related symptom outcomes	At index date^a^ N = 62	2 months n = 45	4 months n = 30	6 months n = 21	8 months n = 10	10 months n = 7	12 months n = 5	At follow-up end date^b^ N = 62
Patients who had at least 1 disease-related symptom, n (%)	62 (100.0)	44 (97.8)	29 (96.7)	20 (95.2)	10 (100.0)	7 (100.0)	4 (80.0)	57 (91.9)
Patients who had at least 1 moderate or severe disease-related symptom, n (%)	48 (77.4)	26 (57.8)	9 (30.0)	3 (14.3)	1 (10.0)	4 (57.1)	1 (20.0)	26 (41.9)
Patients who had at least 1 severe disease-related symptom, n (%)	11 (17.7)	5 (11.1)	2 (6.7)	1 (4.8)	0 (0)	1 (14.3)	0 (0)	11 (17.7)
Patients who received disease-related symptom treatment, n (%)	59 (95.2)	41 (91.1)	22 (73.3)	17 (81.0)	7 (70.0)	7 (100.0)	3 (60.0)	50 (80.6)
Patients who received first-line treatment for LA unresectable or mG/GEJ adenocarcinoma, n (%)	0 (0)	38 (84.4)	28 (93.3)	20 (95.2)	10 (100.0)	6 (85.7)	5 (100.0)	61 (98.4)
Duration of first-line LA unresectable or mG/GEJ adenocarcinoma treatment (months),^c^ mean (SD)	NA	6.3 (3.5)	7.9 (3.8)	8.9 (3.9)	10.9 (4.0)	14.0 (3.1)	13.0 (6.3)	5.4 (3.7)
Follow-up period for disease-related symptoms (months),^d^ mean (SD)	6.1 (3.2)	6.9 (2.8)	8.6 (2.4)	9.4 (2.2)	10.9 (1.3)	11.9 (0.2)	12.0 (0.0)	6.1 (3.2)
Total number of disease-related symptoms, mean (SD)	7.2 (3.3)	6.0 (3.7)	4.2 (3.3)	4.0 (2.5)	4.4 (2.9)	5.4 (3.8)	4.6 (5.2)	4.3 (3.3)
Disease-related symptoms,^e^ n (%)								
Weight loss (*i.e*., loss of ⩾5% since previous visit)	46 (74.2)	16 (35.6)	1 (3.3)	0 (0)	0 (0)	1 (14.3)	0 (0)	9 (14.5)
Abdominal pain/stomach pain	41 (66.1)	26 (57.8)	12 (40.0)	8 (38.1)	5 (50.0)	2 (28.6)	2 (40.0)	22 (35.5)
Anemia/weakness	38 (61.3)	32 (71.1)	21 (70.0)	14 (66.7)	8 (80.0)	6 (85.7)	3 (60.0)	41 (66.1)
Poor appetite	35 (56.5)	19 (42.2)	11 (36.7)	5 (23.8)	4 (40.0)	1 (14.3)	2 (40.0)	25 (40.3)
Epigastric pain^f^	31 (50.0)	4 (8.9)	3 (10.0)	2 (9.5)	2 (20.0)	3 (42.9)	0 (0)	6 (9.7)
Nausea	26 (41.9)	31 (68.9)	18 (60.0)	13 (61.9)	6 (60.0)	4 (57.1)	2 (40.0)	40 (64.5)
Difficulty swallowing (dysphagia)	24 (38.7)	19 (42.2)	7 (23.3)	8 (38.1)	1 (10.0)	3 (42.9)	2 (40.0)	13 (21.0)
Problems eating solid foods	22 (35.5)	13 (28.9)	4 (13.3)	3 (14.3)	0 (0)	2 (28.6)	1 (20.0)	6 (9.7)
Reflux (acid or bile coming into mouth)	22 (35.5)	6 (13.3)	1 (3.3)	1 (4.8)	1 (10.0)	1 (14.3)	0 (0)	5 (8.1)
Worried about their health in the future	21 (33.9)	16 (35.6)	8 (26.7)	5 (23.8)	2 (20.0)	3 (42.9)	2 (40.0)	15 (24.2)
Early satiety	18 (29.0)	11 (24.4)	1 (3.3)	0 (0)	0 (0)	0 (0)	0 (0)	5 (8.1)
Vomiting (with or without blood)	18 (29.0)	16 (35.6)	8 (26.7)	4 (19.0)	4 (40.0)	1 (14.3)	2 (40.0)	14 (22.6)
Thinking about illness	18 (29.0)	16 (35.6)	8 (26.7)	5 (23.8)	2 (20.0)	3 (42.9)	2 (40.0)	15 (24.2)
Heartburn	14 (22.6)	6 (13.3)	0 (0)	0 (0)	1 (10.0)	0 (0)	1 (20.0)	2 (3.2)
Difficulty eating	9 (14.5)	2 (4.4)	2 (6.7)	1 (4.8)	1 (10.0)	1 (14.3)	1 (20.0)	4 (6.5)
Painful swallowing (odynophagia)	8 (12.9)	1 (2.2)	0 (0)	1 (4.8)	0 (0)	1 (14.3)	0 (0)	1 (1.6)
Problems eating liquidized or soft foods	7 (11.3)	3 (6.7)	0 (0)	1 (4.8)	0 (0)	2 (28.6)	0 (0)	2 (3.2)
Cough	6 (9.7)	4 (8.9)	3 (10.0)	2 (9.5)	2 (20.0)	1 (14.3)	1 (20.0)	5 (8.1)
Anorexia	6 (9.7)	2 (4.4)	1 (3.3)	0 (0)	0 (0)	0 (0)	0 (0)	4 (6.5)
Discomfort when eating	5 (8.1)	2 (4.4)	3 (10.0)	2 (9.5)	0 (0)	0 (0)	0 (0)	1 (1.6)
Swelling or fluid buildup in the abdomen	5 (8.1)	2 (4.4)	2 (6.7)	1 (4.8)	0 (0)	0 (0)	0 (0)	5 (8.1)
Problems drinking liquids	4 (6.5)	0 (0)	0 (0)	1 (4.8)	0 (0)	1 (14.3)	0 (0)	0 (0)
Chest pain	4 (6.5)	7 (15.6)	1 (3.3)	2 (9.5)	2 (20.0)	1 (14.3)	1 (20.0)	3 (4.8)
Gastrointestinal bleeding	4 (6.5)	3 (6.7)	2 (6.7)	1 (4.8)	0 (0)	0 (0)	0 (0)	6 (9.7)
Worried about weight being too low	4 (6.5)	0 (0)	0 (0)	0 (0)	0 (0)	0 (0)	0 (0)	0 (0)
Problems with sense of taste	3 (4.8)	4 (8.9)	3 (10.0)	3 (14.3)	2 (20.0)	0 (0)	0 (0)	8 (12.9)
Takes patient a long time to complete their meals	3 (4.8)	2 (4.4)	1 (3.3)	0 (0)	0 (0)	0 (0)	0 (0)	0 (0)
Pain when eating	2 (3.2)	2 (4.4)	0 (0)	0 (0)	0 (0)	0 (0)	0 (0)	0 (0)
Palpable abdominal mass	2 (3.2)	0 (0)	0 (0)	0 (0)	0 (0)	0 (0)	0 (0)	2 (3.2)
Trouble enjoying meals	1 (1.6)	0 (0)	1 (3.3)	0 (0)	0 (0)	0 (0)	0 (0)	1 (1.6)
Dry mouth	0 (0)	0 (0)	0 (0)	0 (0)	0 (0)	0 (0)	0 (0)	1 (1.6)
Choked when swallowing	0 (0)	0 (0)	0 (0)	0 (0)	0 (0)	0 (0)	0 (0)	1 (1.6)
Hemorrhage	0 (0)	1 (2.2)	1 (3.3)	0 (0)	0 (0)	0 (0)	0 (0)	1 (1.6)
Hair loss	0 (0)	1 (2.2)	3 (10.0)	1 (4.8)	1 (10.0)	1 (14.3)	1 (20.0)	2 (3.2)

Patient numbers in column headers indicate the number of patients who had at least the amount of follow-up time indicated for that column. Abbreviations: LA, locally advanced; mG/GEJ, metastatic gastric/gastroesophageal junction; NA, not applicable; SD, standard deviation.

a The index date was defined as the date of diagnosis of LA unresectable or mG/GEJ adenocarcinoma.

b Follow-up end dates were defined as the first-line treatment discontinuation date for 44 patients; the last contact date for 10 patients; 1 year after the index date for 5 patients; both the treatment discontinuation date and last contact date for 2 patients; and the treatment discontinuation date, last contact date, and the date of death for 1 patient.

c Duration of first-line LA unresectable or mG/GEJ adenocarcinoma treatment was calculated among patients who had received first-line treatment for LA unresectable or mG/GEJ adenocarcinoma by this time point.

d Follow-up period for disease-related symptoms was calculated based on the population indicated in the column header, which comprises patients who had at least the amount of follow-up time indicated for that column.

e For patients who had longer than 1 year of follow-up, symptom information was only collected from the index date to 1 year after the index date. Symptoms reported at the index date or follow-up end date are shown.

f Epigastric pain was a category separate from abdominal pain.

**Table 3.** Changes in disease-related symptoms from the index date to the follow-up end date.

Symptoms, treatment use, and severity, n (%)^a^	All patients N = 62
Anemia/weakness	27 (43.5)
Treatment use	
Did not use at index date or follow-up end date	17 (63.0)
Used at index date only	3 (11.1)
Used at follow-up end date only	3 (11.1)
Used at both index and follow-up end date	4 (14.8)
Severity	
Improving	2 (7.4)
Unchanged	22 (81.5)
Worsening	3 (11.1)
Nausea	19 (30.6)
Treatment use	
Did not use at index date or follow-up end date	0 (0)
Used at index date only	0 (0)
Used at follow-up end date only	0 (0)
Used at both index and follow-up end date	19 (100.0)
Severity	
Improving	3 (15.8)
Unchanged	13 (68.4)
Worsening	3 (15.8)
Abdominal pain/stomach pain	17 (27.4)
Treatment use	
Did not use at index date or follow-up end date	0 (0)
Used at index date only	1 (5.9)
Used at follow-up end date only	0 (0)
Used at both index and follow-up end date	16 (94.1)
Severity	
Improving	3 (17.6)
Unchanged	13 (76.5)
Worsening	1 (5.9)
Poor appetite	19 (30.6)
Treatment use	
Did not use at index date or follow-up end date	14 (73.7)
Used at index date only	1 (5.3)
Used at follow-up end date only	4 (21.1)
Used at both index and follow-up end date	0 (0)
Severity	
Improving	3 (15.8)
Unchanged	14 (73.7)
Worsening	2 (10.5)

a Treatment use and severity percentages were calculated among patients with a specific symptom.

**Figure 3.**
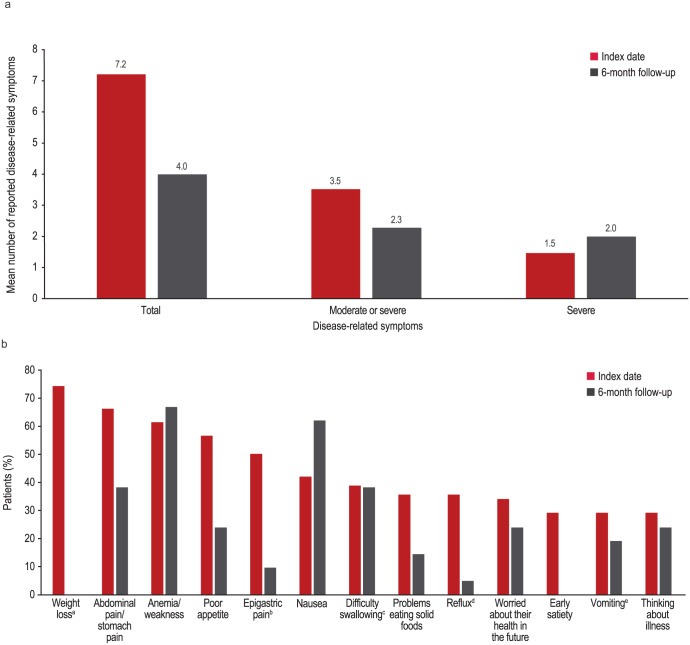
Change in disease-related symptoms from the index date to 6-month follow-up (a) and by disease-related symptoms reported by ⩾25% of patients (b). ^a^Loss of ⩾5% since previous visit. ^b^Epigastric pain was a category separate from abdominal pain. ^c^Dysphagia. ^d^Acid or bile coming into mouth. ^e^With or without blood.

A total of 21 patients were evaluated at the 6-month follow-up, 95.2% of whom continued to have 1 or more disease-related symptoms. At least 1 moderate or severe disease-related symptom was reported by 14.3% of patients. Anemia/weakness (66.7%), abdominal pain/stomach pain (38.1%), and poor appetite (23.8%) remained among the most commonly reported disease-related symptoms. Nausea (61.9%) became a more commonly reported disease-related symptom. In the overall population, fewer disease-related symptoms were reported by patients at the 6-month follow-up compared with the index date (mean [SD] = 4.0 [2.5] at the 6-month follow-up vs 7.2 [3.3] at the index date; Table 2 and Figure 3). The greatest changes in the proportion of patients with disease-related symptoms at the 6-month follow-up compared with the index date were seen for weight loss (0.0% at 6 months vs 74.2% at the index date), epigastric pain (9.5% vs 50.0%), poor appetite (23.8% vs 56.5%), reflux (4.8% vs 35.5%), early satiety (0.0% vs 29.0%), and abdominal pain/stomach pain (38.1% vs 66.1%; Figure 3).

### Healthcare resource utilization

**Figure 4.**
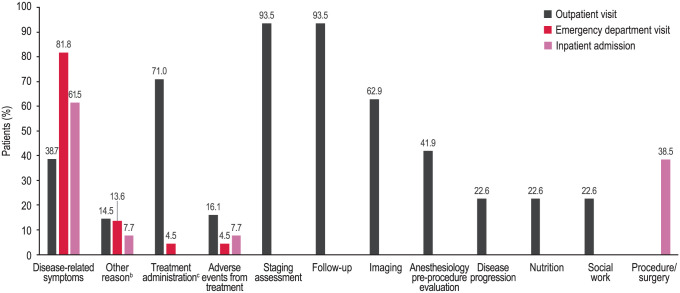
Primary reasons for outpatient visits, emergency department visits, and inpatient admissions.^a^ Abbreviation: CT, computed tomography. ^a^Reasons were reported at the patient level. The visit reason was only counted once, although patients could have multiple visits for the same reason. Percentages for each setting were calculated among patients with at least 1 visit. ^b^Other reasons for outpatient visits were genetic counseling, n = 3; spiritual care, n = 2; COVID-19 testing, n = 2; audiology, n = 1; treatment break request, n = 1; radiation oncology consultation, n = 1; questions about procedure, n = 1; and paracentesis, n = 1. Other reasons for emergency department visits were cerebrovascular accident, n = 1; jejunostomy tube dislodgement, n = 1; and asymptomatic pulmonary embolism found on CT scan, n = 1. Other reason for inpatient admission was cerebrovascular accident, n = 1. ^c^One patient presented to the emergency department for treatment administration.

The mean (SD) duration of follow-up for HRU assessment was 6.5 (4.1) months. All patients had at least 1 outpatient visit during the follow-up period, with a mean of 3.4 (2.1) outpatient visits PPPM. The primary reasons for outpatient visits were staging assessment (93.5%) and follow-up (93.5%; Figure 4). Emergency department visits were reported by 35.5% of patients, with a mean of 0.1 (0.2) visit PPPM. The most common reason for emergency department visits was disease-related symptom treatment (81.8%). Inpatient admissions were reported for 21.0% of patients, with a mean of 0.1 (0.2) visit PPPM. The mean length of hospital stay was 3.4 (1.5) days per admission. The most common reason for inpatient admission was disease-related symptom treatment (61.5%).

Disease-related symptom treatment was received by 95.2% of patients at the index date vs 80.6% of patients at the follow-up end date. Treatment for disease-related symptoms was received at both the index date and the follow-up end date for all patients reporting nausea and for 94.1% of patients reporting abdominal pain/stomach pain. The majority of patients reporting anemia/weakness (63.0%) and most patients reporting poor appetite (73.7%) did not receive treatment at the index date or the follow-up end date.

## Discussion

This retrospective study found that patients with CLDN18.2+, HER2−, LA unresectable or mG/GEJ adenocarcinoma have a high disease-related symptom burden and associated HRU. All patients had at least 1 symptom at the index date. First-line chemotherapy was received by 98.4% of patients. Although patients reported fewer disease-related symptoms at the 6-month follow-up compared with the index date (mean [SD] = 4.0 [2.5] vs 7.2 [3.3]), 95.2% of patients continued to report at least 1 disease-related symptom, with 14.3% of patients reporting at least 1 moderate or severe symptom. Anemia/weakness, abdominal pain/stomach pain, and poor appetite were among the most common disease-related symptoms reported at both the index date and the 6-month follow-up; nausea became a more commonly reported disease-related symptom at the 6-month follow-up. The greatest changes in disease-related symptoms reported at the 6-month follow-up compared with the index date were seen for weight loss (0.0% vs 74.2%), epigastric pain (9.5% vs 50.0%), poor appetite (23.8% vs 56.5%), reflux (4.8% vs 35.5%), early satiety (0.0% vs 29.0%), and abdominal pain/stomach pain (38.1% vs 66.1%). The greatest increase in disease-related symptoms reported at the 6-month follow-up compared with the index date was seen for nausea (61.9% vs 41.9%). The severity level reported for anemia/weakness, abdominal pain/stomach pain, poor appetite, and nausea did not change from the index date to the follow-up end date. Patients had a mean (SD) of 3.4 (2.1) outpatient visits PPPM. The mean (SD) number of emergency department visits and inpatient admissions PPPM was 0.1 (0.2) for each. Disease-related symptoms were the most common reason for emergency department visits (81.8%) and inpatient admission (61.5%). Disease-related symptom treatment was received by 95.2% and 80.6% of patients at the index date and follow-up end date, respectively. Treatment use occurred most often at both the index date and the follow-up end date for nausea and abdominal pain/stomach pain. Most patients who reported anemia/weakness (63.0%) and poor appetite (73.7%) did not use treatment at the index date or the follow-up end date.

Although the published literature specifically characterizing symptom burden and HRU in patients with CLDN18.2+ LA unresectable or mG/GEJ adenocarcinoma is lacking, studies in broader G/GEJ cancer populations, including patients with LA unresectable or mG/GEJ adenocarcinoma, report high symptom burden and HRU.^5,14^ Common disease-related symptoms of LA unresectable or mG/GEJ adenocarcinoma include negative effects on energy level (*i.e.*, fatigue, tiredness, or drowsiness), poor appetite, pain, nausea, vomiting, dysphagia, weight loss, anxiety, and depression.^3,5,6^ Almost 50% of adult patients scheduled to undergo gastrectomy for treatment of gastric cancer report moderate-to-severe symptoms, the most prevalent being fatigue, pain, poor appetite, and constipation.^5^ Anemia/weakness and poor appetite, 2 of the most common disease-related symptoms reported at both the index date and the follow-up end date in this study, are associated with poor prognosis for patients with LA unresectable or mG/GEJ adenocarcinoma.^3,8,24^

Poor appetite can lead to weight loss.^3^ Increased symptom burden is also associated with poorer overall survival rates and may play a role in determining overall survival in patients with mG/GEJ cancer.^25^ Overall survival rates in patients with gastric cancer who received first-line chemotherapy were found to be significantly worse among those who had weight loss (defined as loss of >5% of body weight or loss of >2% of body weight with a body mass index of <20 kg/m^2^ within the last 6 months) compared with those who did not have weight loss after starting chemotherapy.^8^ Patients with gastric cancer and a low body mass index have also been reported to have significantly worse overall survival rates compared with patients with gastric cancer who have a high body mass index.^7^

Symptom frequency has been reported as unchanged or worsened with the initiation of chemotherapy.^12^ For example, compared with pre-chemotherapy symptom reports, a greater proportion of patients experienced moderate-to-severe lethargy after initiating chemotherapy for gastrointestinal malignancies, with these proportions not declining until 12 months after chemotherapy initiation.^12^ Symptom severity also increases in the last 6 months of life, with tiredness and poor appetite being among the most common symptoms reported by patients.^3^ An increase in the frequency and severity of symptoms can lead to increased HRU. A prior retrospective claims analysis, with a median follow-up of 8.6 months, performed between October 2016 and December 2019 for patients with HER2− G/GEJ cancer found that most patients had at least 1 outpatient visit in the study period with a mean of approximately 3 to 4 outpatient visits per month.^14^

The results of this study provide further evidence supporting the high symptom burden and HRU associated with CLDN18.2+, HER2−, LA unresectable or mG/GEJ adenocarcinoma. Because first-line chemotherapy treatment had not been started at the index date, the symptoms reported at that time were more likely to be disease-related as opposed to treatment-related. The 6-month follow-up was used for comparison with the study index date for disease-related symptom reporting because 6 months is a commonly scheduled time for clinical evaluation, the number of patients being followed (n = 21) was sufficient for meaningful observations, and selecting a fixed time point avoided variations in the follow-up end date for each patient. The change in the mean number of symptoms reported and reported symptom severity observed over the short follow-up time of this study may have resulted from several factors. First, the patient population analyzed can be assumed to be relatively functional because 91.9% of patients had an ECOG performance status of 1, and 98.4% of patients received first-line treatment. Second, first-line chemotherapy did provide some disease-related symptom control. Finally, patients may also have increased symptom tolerability, resulting in reduced reporting of symptoms over time. However, a majority of patients in this study continued to experience disease-related symptoms despite receiving standard-of-care chemotherapy, and HRU for disease-related symptom management continued to occur. Given the progressive nature of LA unresectable or mG/GEJ adenocarcinoma and lack of curative treatments, disease-related symptom burden and HRU may continue to increase with time after diagnosis.^14,26^

### Limitations

Limitations of this study include the collection of data from a single center; the potential for inaccurate or incomplete data recorded in the electronic medical record; a small sample size, which limited assessment to descriptive statistics; a lack of racial and ethnic diversity within the sample; the potential for overlap of reported symptoms and symptom categories; and the potential sponsor bias. Notably, symptoms described at the index visit occurred prior to the initiation of chemotherapy and therefore are likely to reflect disease-related symptoms. However, symptoms described at visits after the index date are more difficult to categorize as disease-related symptoms, treatment-related symptoms, or both. Although the limitations of the data collected may affect the generalizability of the findings, results are in line with prior reports on disease-related symptoms of G/GEJ cancer, including LA unresectable or mG/GEJ adenocarcinoma, and associated HRU.^5,14^

## Conclusion

This retrospective analysis showed substantial disease-related symptom burden and high HRU for LA unresectable or mG/GEJ adenocarcinoma despite standard-of-care chemotherapy, indicating an unmet need for better treatment options. The development of effective and well-tolerated treatments may help reduce disease-related symptom burden and associated HRU, potentially allowing patients to tolerate further disease treatment and improving quality of life.
